# Performance of the Enhanced Liver Fibrosis Test to Estimate Advanced Fibrosis Among Patients With Nonalcoholic Fatty Liver Disease

**DOI:** 10.1001/jamanetworkopen.2021.23923

**Published:** 2021-09-16

**Authors:** Zobair M. Younossi, Sean Felix, Thomas Jeffers, Elena Younossi, Fatema Nader, Huong Pham, Arian Afendy, Rebecca Cable, Andrei Racila, Zahra Younoszai, Brian P. Lam, Pegah Golabi, Linda Henry, Maria Stepanova

**Affiliations:** 1 Betty and Guy Beatty Center for Integrated Research, Inova Health System, Falls Church, Virginia; 2 Department of Medicine, Inova Health System, Falls Church, Virginia; 3 Center for Liver Diseases, Department of Medicine, Inova Fairfax Medical Campus, Falls Church, Virginia; 4 Center for Outcomes Research in Liver Diseases, Washington, District of Columbia

## Abstract

**Importance:**

The most important surrogate for increased risk of adverse clinical outcomes among patients with nonalcoholic fatty liver disease (NAFLD) is the patient’s stage of liver fibrosis. There is a significant barrier to risk-stratifying patients in clinical practice owing to the need for liver biopsy.

**Objective:**

To determine the performance of the enhanced liver fibrosis (ELF) test as a noninvasive test for assessment of liver fibrosis among patients with NAFLD.

**Design, Setting, and Participants:**

This retrospective cross-sectional study was conducted among patients recruited from a large, community-based hospital system’s outpatient liver clinic from 2001 to 2020. Patients with NAFLD defined as steatosis greater than 5% without evidence of other liver disease or excessive alcohol use were included. Data were analyzed from August 2020 through February 2021.

**Intervention:**

Enhanced liver fibrosis score was calculated.

**Main Outcomes and Measures:**

Advanced fibrosis was identified by liver biopsy or transient elastography.

**Results:**

Among 829 patients with NAFLD, the mean (SD) age was 53.1 (14.0) years, there were 363 (43.8%) men, 294 patients (35.5%) had type 2 diabetes, and the mean (SD) fibrosis-4 (fib-4) score was 1.34 (0.97). There were 463 patients with liver biopsy, among whom 113 individuals (24.4%) had bridging fibrosis or cirrhosis; among 462 patients with transient elastography data, 79 individuals (17.1%) had liver stiffness results of 9.6 kPa or more (ie, advanced fibrosis). Patients with advanced fibrosis had statistically significantly increased mean (SD) ELF scores compared with patients without advanced fibrosis as determined by biopsy (10.1 [1.3] vs 8.6 [1.0]; P < .001) or transient elastography (10.0 [1.1] vs 9.0 [0.8]; *P* < .001). Among all patients with NAFLD, the area under the receiver operating characteristic curve (AUROC) for ELF in identifying patients with advanced fibrosis was 0.81 (95% CI, 0.77-0.85) for patients diagnosed by biopsy and 0.79 (95% CI, 0.75-0.82) for those diagnosed by transient elastography. Performance of the ELF score was similar among patients with NAFLD who were aged 65 years or older (AUROC, 0.74; 95% CI, 0.58-0.87) or had type 2 diabetes (AUROC, 0.78; 95% CI, 0.71-0.84). The combination of an ELF score of 7.2 or greater with a fib-4 score of 0.74 or greater was associated with a negative predictive value of 95.1% (95% CI, 91.8%-98.4%) and a sensitivity of 92.5% (95% CI, 87.4%-97.5%), which can reliably rule out advanced fibrosis. An ELF score of 9.8 or greater with a fib-4 score of 2.9 or greater was associated with a positive predictive value of 95.0% (95% CI, 85.5%-100%) and a specificity of 99.7% (95% CI, 99.1%-100%), which can be used to rule in advanced fibrosis.

**Conclusions and Relevance:**

These findings suggest that the ELF test performs well in identifying patients with NAFLD who are at increased risk of advanced fibrosis and that this test combined with fib-4 score may be reliably used in clinical practice to assess the presence or absence of advanced fibrosis among patients with NAFLD.

## Key Points

**Question:**

Can noninvasive tests be used to accurately rule in and rule out advanced fibrosis among patients with nonalcoholic fatty liver disease (NAFLD)?

**Findings:**

This cross-sectional study of 829 patients with NAFLD found that the noninvasive enhanced liver fibrosis (ELF) test can be used to estimate advanced fibrosis among patients with NAFLD with an area under the receiver operator characteristic curve of 0.81, with a similar performance observed among patients with NAFLD who had diabetes or were age 65 years or older and in an independent validation set. Different combinations of cutoff values to rule in advanced fibrosis were associated with a specificity of 99.7% and positive predictive value of 95.0% or a sensitivity of 92.5% and negative predictive value of 95.0%.

**Meaning:**

These findings suggest that the ELF test can be used in gastroenterology, endocrinology, and primary care practices to identify patients with increased risk of nonalcoholic steatohepatitis who would require aggressive treatment.

## Teaser

This cross-sectional study investigates use of the noninvasive enhanced liver fibrosis test to estimate advanced fibrosis among patients with nonalcoholic fatty liver disease.

## Introduction

As the prevalence of obesity increases worldwide, so does the prevalence of nonalcoholic fatty liver disease (NAFLD) and its complications.^1,2^ In this context, the stage of fibrosis seems to be a factor associated with mortality.^3^ Therefore, identifying patients with NAFLD who have advanced fibrosis is of clinical importance.^4,5,6,7^ Furthermore, ruling out advanced fibrosis noninvasively is also clinically relevant. In fact, although liver biopsy is considered a criterion standard for determination of steatohepatitis and stage of fibrosis, it has many drawbacks, including an invasive nature, sampling issues, and observer variability in pathologic assessment.^8,9^ Therefore, efforts have been ongoing to develop noninvasive tests (NITs) that may be used in clinical practice for assessment of NAFLD-associated fibrosis.^10,11^

In general, NITs can be divided into 3 categories: (1) clinical tools that use routinely collected laboratory and clinical parameters to calculate a risk score, among which the most commonly used are fibrosis-4 (fib-4) index score, NAFLD fibrosis score (NFS), and aspartate aminotransferase–platelet ratio index score; (2) radiologic tests that assess liver stiffness, among which the most commonly used method is transient elastography (TE), while other liver stiffness–assessment methods include shear elastography, acoustic radiation force impulse imaging, and magnetic resonance elastography; and (3) blood-based tests that use markers of fibrogenesis and cytolysis, among which enhanced liver fibrosis (ELF) is currently approved in Europe. However, each test used in isolation has limitations, which include large gray zones hindering accurate decision-making.^10,11^ Additionally, some tests, such as TE, may not be widely available. To improve performance of these NITs, algorithms have been developed to combine multiple NITs and improve their ability to assess the stage of fibrosis among patients with NAFLD.^11^

The ELF test is a noninvasive blood-derived panel of biomarkers consisting of 3 components: type III procollagen peptide, hyaluronic acid, and tissue inhibitor of metalloproteinase-1.^12^ A 2020 meta-analysis^13^ of the performance of the ELF test found that it had high sensitivity but limited specificity to exclude advanced fibrosis at low cutoff values, especially in low-prevalence settings. On the other hand, a 2021 systematic review^14^ found that ELF had excellent performance in detecting advanced fibrosis among patients with NAFLD (area under the receiver operating characteristic curve [AUROC], 0.78-0.97) and evidence of good diagnostic performance for detecting cirrhosis (AUROC, 0.85-0.92). Another study of a small sample of patients with nonalcoholic steatohepatitis (NASH) diagnosed by liver biopsy reported that ELF performed excellently in diagnosing advanced fibrosis compared with other noninvasive tests, including FibroMeterV2G (Echosens), FibroMeterV3G (Echosens), FibroScan (Echosens), NFS, and fib-4.^15^ The Stellar clinical trials data were evaluated for the ability of noninvasive tests, including ELF, to predict advanced fibrosis and found that ELF performed well; however, because most patients in the population had advanced fibrosis, the ability to rule out advanced fibrosis was hindered.^16^ In this study, we aimed to investigate the accuracy of ELF in ruling in and out the presence of advanced fibrosis using a large sample of patients with NAFLD enrolled in a real-world hepatology practice.

## Methods

This cross-sectional study protocol was approved by the Inova Health Systems institutional review board. Written informed consent was obtained for all participants. This manuscript is reported following the Strengthening the Reporting of Observational Studies in Epidemiology (STROBE) reporting guideline for cross-sectional studies.

### Patient Population

Patients with liver disease were referred to our outpatient liver clinic by other clinicians, including those in primary care, endocrinology, and gastroenterology. Patients identified as having NAFLD and having attended our clinic from 2001 to 2020 were eligible for this study. Clinical and laboratory data along with serum samples were collected for each patient after obtaining informed consent. We included 1 record per patient. Patients with evidence of excessive alcohol use (ie, ≥10 g/d) or other causes of liver disease (eg, hepatitis B, hepatitis C, or autoimmune liver disease) and those receiving treatment with peroxisome proliferator–activated receptor-γ agonists were excluded. Race and ethnicity were self-reported, and these data were collected because there is some racial and ethnic disparity in NAFLD and NASH severity.^17^

Patients included in this study had a liver biopsy or a liver stiffness assessment by TE; the choice of a diagnostic method was made in the course of the patient’s clinical care based on the clinician’s recommendation and not based on enrollment in this study. Liver biopsies were read by a single hepatopathologist. Liver fibrosis was graded from 0 to 4 using NASH Clinical Research Network criteria^18^; advanced fibrosis was defined as stage 3 or 4 (ie, bridging fibrosis or cirrhosis). TE was administered using FibroScan Mini 430 (Echosens); per manufacturer guidelines, 10 or more adequate measurements were obtained, and the median stiffness and controlled attenuation parameter (CAP) scores were automatically calculated. Patients with liver stiffness of 9.6 kPa or more were presumed to have advanced hepatic fibrosis.

### ELF Score

Frozen serum samples that had been collected at the time of liver biopsy or TE were used for determination of ELF scores, which were calculated using an Advia Centaur XP analyzer (Siemens Healthineers). While the rest of the clinical and laboratory parameters used in this study were recorded at the time of the original clinic visit, ELF scores were measured later using frozen serum samples. The samples were snap frozen at collection, continuously stored in −80 °C freezers in our clinic, and evaluated for quality control by Siemens laboratory personnel who ran the assays. To date, there is no data regarding maximum storage time for measuring ELF score in historic samples or whether sample degradation with increased storage time is associated with stability of ELF components.^12^ In this study, we found no correlation of time spent in storage with ELF score: Pearson *r* = −0.04 (*P* = .21) with adjustment for the presence of advanced fibrosis. In addition to ELF, we used laboratory parameters collected at the time of the original clinic visit (ie, alanine aminotransferase [ALT], aspartate aminotransferase [AST], and albumin levels and platelet count) to calculate fib-4 and NFS scores.

### Statistical Analysis

The 2 study outcomes were advanced fibrosis by liver biopsy and by TE. Variables were summarized as mean (SD) or numbers with percentages. Comparisons between groups of patients with and without advanced fibrosis by either definition were performed by nonparametric Mann-Whitney tests or χ^2^ tests. Correlations between ELF scores and other continuous parameters were assessed using Pearson correlation coefficients. Unless otherwise noted, we used 2-tailed hypothesis tests, and *P* values < .05 were considered statistically significant.

The accuracy of ELF for estimating the presence of advanced fibrosis was assessed with receiver operating characteristic curves, and area under the receiver operating characteristic curve (AUROC) was calculated with 95% CIs. Preselected ELF cutoffs from the literature were used to assess sensitivity, specificity, positive predictive value (PPV), and negative predictive value (NPV): 9.8 to rule out advanced fibrosis and 11.3 to rule in advanced fibrosis.^19^ To examine the validity of these rules in another external sample, the same calculations were performed in a nonoverlapping testing sample that included deidentified data from patients with biopsy-confirmed NASH enrolled in a previously described registry of our patients seen in other settings.^20^ Patients with and without advanced fibrosis from the registry for whom fib-4 and ELF scores were available were randomly sampled, with the prevalence of advanced fibrosis set similar to that seen in the original study sample. In addition, with the aim of potentially improving performance of ELF when used in combination with another score, we ran dynamic cutoff searches for combinations of 2 noninvasive tests (ie, ELF and fib-4 or NFS and fib-4) by recording accuracy metrics for all possible combinations of cutoffs for 2 scores and choosing combinations with accuracy metrics that met prespecified criteria. In this 2-dimensional search, a criterion that would return the highest sensitivity for a prespecified PPV or the highest specificity for a prespecified NPV would be chosen. Because the sample of patients enrolled in our liver clinic may be associated with a referral bias, we additionally ran a sensitivity analysis presuming a decreased prevalence of advanced fibrosis in the patient population, which may be expected in primary care setting.

All analyses were run using SAS statistical software version 9.4 (SAS Institute). Data were analyzed from August 2020 through February 2021.

## Results

Among 829 patients with NAFLD included (mean [SD] age, 53.1 [14.0] years; 363 [43.8%] men), 463 patients had a liver biopsy and 462 patients had TE (Table 1; eTable 1 in the Supplement). There were 561 White individuals (67.7%), 111 Black individuals (13.4%), 51 Asian individuals (13.4%), 66 Hispanic individuals (8.0%), and 38 individuals with other racial or ethnic backgrounds (4.6%). Among all patients, 294 individuals (35.5%) had type 2 diabetes, 522 individuals (67.4%) had hyperlipidemia, and 505 individuals (63.4%) had hypertension. The mean (SD) was 36.5 (9.7) for body mass index (BMI; calculated as weight in kilograms divided by height in meters squared), 42.8 (35.7) U/L for ALT level (to convert to microkatals per liter, multiply by 0.0167), and 33.7 (25.0) U/L for AST level (to convert to microkatals per liter, multiply by 0.0167). The mean (SD) ELF score was 9.03 (1.10), with 649 patients (78.3%) with an ELF of less than 9.8, 148 patients (17.8%) with an ELF from 9.8 to less than 11.3, and 32 patients (3.9%) with an ELF of 11.3 or more.

**Table 1.** Clinical and Demographic Characteristics of Patients

Characteristic^a^	Patients, No. (%) (N = 829)
Age, mean (SD), y	53.1 (14.0)
Sex	
Men	363 (43.8)
Women	466 (56.2)
Race and ethnicity	
White	561 (67.7)
Black	111 (13.4)
Asian	51 (6.2)
Hispanic	66 (8.0)
Other^b^	38 (4.6)
BMI, mean (SD)	36.5 (9.7)
Type 2 diabetes	294 (35.5)
Hyperlipidemia	522 (67.4)
Hypertension	505 (63.4)
Aminotransferase, mean (SD), U/L	
Alanine	42.8 (35.7)
Aspartate	33.7 (25.0)
Platelet count, mean (SD), ×10^3^/μL	242.6 (73.7)
TE, mean (SD), kPa	7.73 (6.29)
ELF score	
Mean (SD)	9.03 (1.10)
Median (IQR)	8.9 (8.3 to 9.6)
Fib-4 score	
Mean (SD)	1.34 (0.97)
Median (IQR)	1.08 (0.70 to 1.70)
NAFLD fibrosis score	
Mean (SD)	−0.83 (1.75)
Median (IQR)	−0.93 (−1.98 to 0.29)
Biopsy available	463 (55.9)
TE available	462 (55.7)

Abbreviations: BMI, body mass index (calculated as weight in kilograms divided by height in meters squared); ELF, enhanced liver fibrosis; fib-4, fibrosis-4; IQR, interquartile range; NAFLD, nonalcoholic fatty liver disease; TE, transient elastography.

SI conversion factors: To convert alanine aminotransferase and aspartate aminotransferase to microkatals per liter, multiply by 0.0167; platelet count to ×10^9^/L, multiply 1.0.

^a^ The same parameters summarized separately for patients with liver biopsy and with TE are shown in eTable 1 in the Supplement.

^b^ Other race and ethnicity included patients who indicated being of more than 1 race or ethnicity, Native Hawaiian, Pacific Islander, American Indian, or Alaska Native, or who declined to disclose this information.

Among all patients, ELF was positively correlated with ALT level (Pearson *r* = 0.18; *P* < .001) and AST level (ρ = 0.30; *P* < .001) (eFigure in the Supplement). However, ELF was negatively correlated with platelet count (ρ = −0.33; *P* < .001) and BMI (ρ = −0.16; *P* < .001) (eFigure in the Supplement).

**Figure 1.**
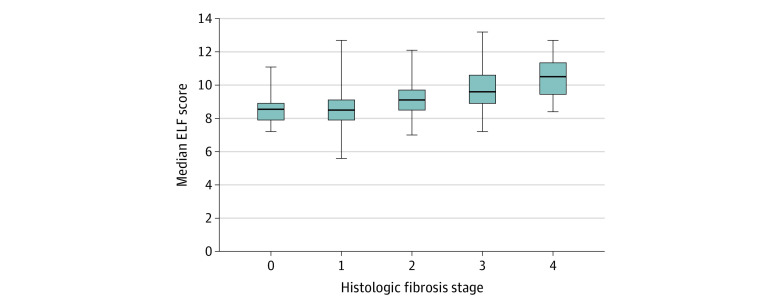
Enhanced Liver Fibrosis (ELF) Score by Histologic Fibrosis Stage Horizontal lines indicate medians; boxes, interquartile ranges; whiskers, 95% CIs. The Clinical Research Network scoring system was used for staging.

Among patients with liver biopsy, 113 individuals (24.4%) had histologic evidence of bridging fibrosis or cirrhosis, as determined by the study pathologist. Patients with advanced fibrosis had statistically significantly increased mean (SD) ELF scores compared with patients without advanced fibrosis as determined by biopsy (10.1 [1.3] vs 8.6 [1.0]; *P* < .001). ELF was correlated with semiquantitative fibrosis stage (ρ = 0.53; *P* < .001) (Figure 1). Under the assumption of a linear association, the median (SE) estimated increase in ELF score with each additional histologic fibrosis stage was 0.55 (0.04). Owing to clear violation of linearity as shown in Figure 1, stages 0 and 1 were pooled together, with a median (SE) increase of 0.64 (0.05).

Among patients with TE data, mean (SD) liver stiffness was 7.7 (6.3) kPa and mean (SD) CAP was 305 (58) dB/m. Of these patients, 79 individuals (17.1%) had liver stiffness of 9.6 kPa or more and were presumed to have advanced fibrosis. Similar to the results for advanced fibrosis by biopsy, patients with NAFLD with advanced fibrosis by TE had increased mean (SD) ELF compared with patients without advanced fibrosis by TE (10.0 [1.1] vs 9.0 [0.8]; *P* < .001). Correlation of ELF with liver stiffness was similar to that with semiquantitative histologic assessment at ρ = 0.46 (*P* < .001) (eFigure in the Supplement). In a linear regression model, a median (SE) increase in ELF of 0.14 (0.01) would be expected for each additional 2 kPa of liver stiffness.

**Figure 2.**
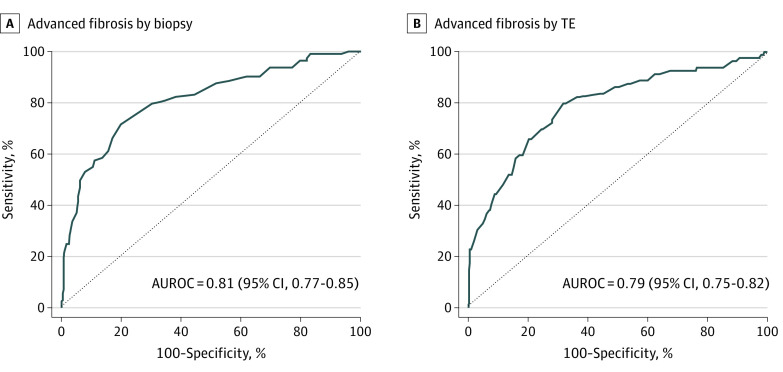
Estimation of Advanced Fibrosis With Enhanced Liver Fibrosis Advanced fibrosis by transient elastography (TE) indicates liver stiffness of 9.6 kPa or greater; dotted line, an area under the receiver operating characteristic curve (AUROC) of 0.5, corresponding to a no predictive power.

Among all patients with NAFLD, the AUROC for ELF in identifying patients with advanced fibrosis was 0.81 (95% CI, 0.77-0.85) by biopsy and 0.79 (95% CI, 0.75-0.82) by TE (Table 2, Figure 2; eTable 2 and eTable 3 in the Supplement). Performance of the ELF score was similar among patients with NAFLD who were aged 65 years or older (AUROC, 0.74; 95% CI, 0.58-0.87) or had type 2 diabetes (AUROC, 0.78; 95% CI, 0.71-0.84) (eTable 4 and eTable 5 in the Supplement).

**Table 2.** Accuracy of Estimating Advanced Fibrosis With ELF Score

Score cutoff value	Performance in predicting advanced fibrosis, % (95% CI)^a^			
	Among patients with liver biopsy		Among patients with TE	
	All patients (n = 463)	Patients with type 2 diabetes (n = 161)	All patients (n = 462)	Patients with type 2 diabetes (n = 177)
Patients with advanced fibrosis, No. (%)	113 (24.4)	73 (45.3)	79 (17.1)	55 (31.1)
AUROC curve (95% CI)	0.81 (0.77-0.85)	0.78 (0.71-0.84)	0.79 (0.75-0.82)	0.80 (0.73-0.86)
**ELF cutoff = 9.8**				
Sensitivity	57.5 (48.4-66.6)	60.3 (49.1-71.5)	58.2 (47.3-69.1)	72.7 (61.0-84.5)
Specificity	88.9 (85.6-92.2)	86.4 (79.2-93.5)	84.1 (80.4-87.7)	75.4 (67.8-83.1)
PPV	62.5 (53.2-71.8)	78.6 (67.8-89.3)	43.0 (33.6-52.4)	57.1 (45.6-68.7)
NPV	86.6 (83.1-90.2)	72.4 (63.8-80.9)	90.7 (87.7-93.7)	86.0 (79.4-92.6)
**ELF cutoff = 11.3**				
Sensitivity	19.5 (12.2-26.8)	19.2 (10.2-28.2)	17.7 (9.3-26.1)	23.6 (12.4-34.9)
Specificity	99.1 (98.2-100)	100 (95.9-100)	99.5 (98.8-100)	100 (97.0-100)
PPV	88.0 (75.3-100)	100 (76.8-100)	87.5 (71.3-100)	100 (75.3-100)
NPV	79.2 (75.4-83.0)	59.9 (51.9-67.8)	85.4 (82.2-88.7)	74.4 (67.7-81.1)
**ELF ≥ 9.8 and fib-4 ≥ 2.9**				
Sensitivity	17.9 (10.6-25.2)	19.4 (10.3-28.6)	17.1 (8.6-25.6)	22.2 (11.1-33.3)
Specificity	99.7 (99.1-100)	100 (95.7-100)	99.2 (98.2-100)	99.2 (97.5-100)
PPV	95.0 (85.5-100)	100 (76.8-100)	81.3 (62.1-100)	92.3 (77.8-100)
NPV	78.5 (74.5-82.5)	59.2 (51.1-67.2)	85.1 (81.7-88.5)	73.4 (66.5-80.3)
**ELF ≥ 7.2 and fib-4 ≥ 0.74** ^**b**^				
Sensitivity	92.5 (87.4-97.5)	95.8 (91.2-100)	90.8 (84.3-97.3)	92.6 (85.6-99.6)
Specificity	48.7 (43.3-54.2)	36.9 (26.6-47.2)	15.5 (11.7-19.2)	13.7 (7.5-19.9)
PPV	37.6 (31.7-43.4)	56.6 (47.8-65.4)	18.4 (14.5-22.3)	33.1 (25.6-40.6)
NPV	95.1 (91.8-98.4)	91.2 (81.6-100)	88.9 (81.1-96.7)	80.0 (62.5-97.5)

Abbreviations: AUROC, area under the receiver operating characteristic; ELF, enhanced liver fibrosis; fib-4, fibrosis-4; NPV, negative predictive value; PPV, positive predictive value; TE, transient elastography.

^a^ The prevalence of advanced fibrosis in each subgroup is shown in brackets. Comparison with other noninvasive tests is shown in eTable 3 in the Supplement. A sensitivity analysis for hypothetical populations with decreased rates of advanced fibrosis is shown in eTable 2 in the Supplement. A comparison of accuracy among patients with and without type 2 diabetes separately is shown in eTable 4 in the Supplement and by age group in eTable 5 in the Supplement.

^b^ A negative rule that may be used to rule out advanced fibrosis is as follows: ELF less than 7.2 or fib-4 less than 0.74.

Using ELF scores of 9.8 or greater for estimation of advanced fibrosis among patients with NAFLD, patients with biopsy had a sensitivity of 57.5% (95% CI, 48.4%-66.6%), a specificity of 88.9% (95% CI, 85.6%-92.2%), a PPV of 62.5% (95% CI, 53.2%-71.8%), and an NPV of 86.6% (95% CI, 83.1%-90.2%), while patients with TE had a sensitivity of 58.2% (95% CI, 47.3%-69.1%), a specificity of 84.1% (95% CI, 80.4%-87.7%), a PPV of 43.0% (95% CI, 33.6%-52.4%), and an NPV 90.7% (95% CI, 87.7%-93.7%). On the other hand, using ELF scores of 11.3 or greater, patients with biopsy had a sensitivity of 19.5% (95% CI, 12.2%-26.8%), a specificity of 99.1% (95% CI, 98.2%-100%), a PPV of 88.0% (95% CI, 75.3%-100%), and an NPV of 79.2% (95% CI, 75.4%-83.0%), while patients with TE had a sensitivity of 17.7% (95% CI, 9.3%-26.1%), a specificity of 99.5% (95% CI, 98.8%-100%), a PPV of 87.5% (95% CI, 71.3%-100%), and an NPV of 85.4% (95% CI, 82.2%-88.7%) (Table 2). Performance of ELF according to the same criteria was similar among all patients with NAFLD and patients with type 2 diabetes (Table 2).

After running a dynamic cutoff search for the 2 NITs that would return superior PPV or NPV for identifying histologic advanced fibrosis in our sample, we found that adding fib-4 values of 1.24 or more to the previously described cutoff of 11.3 for ELF was associated with improvement in PPV from 88.0% to 91.3% (95% CI, 79.8%-100%) while keeping sensitivity at approximately 20% (19.8% [95% CI, 12.2%-27.4%]). We found that a rule setting ELF at 9.8 or greater and fib-4 at 2.9 or greater was associated with a PPV of 95.0% (95% CI, 85.5%-100%), with a sensitivity of 17.9% (95% CI, 10.6%-25.2%), a specificity of 99.7% (95% CI, 99.1%-100%), and an NPV of 78.5% (95% CI, 74.5%-82.5%). Similarly, with a rule setting ELF at 7.2 or more and fib-4 at 0.74 or more was associated with an NPV of 95.1% (95% CI, 91.8%-98.4%), with a sensitivity of 92.5% (95% CI, 87.4-97.5), a specificity of 48.7% (95% CI, 43.3%-54.2%), and a PPV of 37.6% (95% CI, 31.7%-43.4%). Based on the latter result, a combination to rule out advanced fibrosis could be rewritten as ELF less than 7.2 or fib-4 less than 0.74 (Table 2).

In a sensitivity analysis, we found that a modeled decrease in the prevalence of advanced fibrosis in the population was associated with improved NPV (97.4% in a population with 15% advanced fibrosis, 99.2% in a population with 5% advanced fibrosis), while PPV remained as high as 75.8% in a population with 5% prevalence of advanced fibrosis and 91.3% in a population with 15% prevalence (eTable 2 in the Supplement). In addition, we ran a similar cutoff search for a combination of NFS and fib-4 levels and found that the highest possible PPV was 93.3% (95% CI, 80.7%-100%) with a sensitivity of 13.5% (95% CI, 6.9%-20.0%) for the rule setting fib-4 at 3.33 or more and NFS at 0.63 or more, while an NPV of 96.9% (95% CI, 93.4%-100%) was observed with a specificity of 29.8% (95% CI, 24.7%-34.9%) for the rule setting fib-4 at 0.56 or more and NFS at −3.9 or more.

The external testing sample consisted of 912 patients with biopsy-confirmed NASH, including 240 patients (26.3%) with advanced fibrosis and 672 patients (73.7%) without advanced fibrosis (eTable 6 in the Supplement). In this sample, a rule setting ELF at 9.8 or more and fib-4 at 2.9 or more was associated with a sensitivity of 25.1% (95% CI, 19.6%-30.6%), a specificity of 97.9% (95% CI, 96.8%-99.0%), a PPV of 81.1% (95% CI, 72.2%-90.0%), and an NPV of 78.6% (95% CI, 75.8%-81.4%). A rule setting ELF at 7.2 or more and fib-4 at 0.74 or more was associated with a sensitivity of 98.3% (95% CI, 96.7%-100%), a specificity of 18.3% (95% CI, 15.4%-21.3%), a PPV of 30.0% (95% CI, 26.8%-33.2%), and an NPV of 96.9% (95% CI, 93.8-99.9%) (eTable 2 in the Supplement).

## Discussion

In this cross-sectional study, we used a large sample of patients with an established diagnosis of NAFLD to assess the performance of ELF score in ruling in or ruling out the presence of advanced fibrosis. We found that ELF by itself performed well as shown by AUROCs for advanced fibrosis. In addition, regardless of ELF cutoff scores used, ELF score was associated with high specificity scores for advanced fibrosis. In fact, the specificity approached 100% when an ELF cutoff of 11.3 was used, regardless of method of fibrosis ascertainment.

Despite these results, the NPV of ELF alone did not exceed 85% in our sample, in which the prevalence of advanced fibrosis of was approximately 25% among patients with liver biopsy, which is close to that seen by hepatologists in their daily practice,^21^ and evidence from prior studies suggests that the use of 2 or more NITs may be associated with improved diagnostic accuracy. Therefore, we added fib-4 to investigate whether this addition was associated with improved performance.^11,22^ We selected fib-4 because of its simple formula, requiring routinely collected laboratory parameters, which makes it one of the most commonly used NITs in clinical practice.^11^ We found that a combination of an ELF cutoff of 7.2 and a fib-4 score cutoff of 0.74 was associated with an NPV of 95.1% with a sensitivity of 92.5%. These results suggest that the presence of advanced fibrosis can be ruled out among patients with 1 of those scores falling below those thresholds. On the other hand, the combination of cutoffs of 9.8 and 2.9 for ELF and fib-4, respectively, was associated with a PPV of 95.0% with a specificity of 99.7%. It is important to note that these performance metrics were reproduced in an independent sample of patients with NASH who had been enrolled in our registry and in subgroups of older patients and those with type 2 diabetes. The latter group is clinically relevant to primary care clinicians for those patients given that most individuals with diabetes also have NAFLD.^23^ If additional independent validation is completed, we recommend that a combination of ELF and easily calculated fib-4 may provide the best performance in ruling in or out advanced fibrosis among patients with NAFLD.

In the clinical setting, the use of an ELF and fib-4 combination to rule out advanced fibrosis may provide valuable information to health care professionals to identify patients with NAFLD who are not likely to be at risk of adverse hepatic outcomes; rather, these patients may be followed up by their primary care practitioners to manage their cardiometabolic risks.^10,11^ Additionally, the use of these tests to detect advanced fibrosis with high PPV without the need to perform a liver biopsy may be valuable because patients with increased likelihood of advanced fibrosis may be further linked to gastroenterology or hepatology care and receive more aggressive treatment. Additionally, these patients may be considered for clinical trials of new pharmacologic agents for NASH. It is also important to note that patients with NAFLD and cirrhosis are at risk for hepatocellular carcinoma and other complications, so additional screening programs may need to be considered.^24,25,26^

### Limitations and Strengths

This study has several limitations, including a limited sample size of patients with liver biopsy. Notably, because TE itself is a noninvasive test, its accuracy may be a limiting factor. However, the performance of ELF in estimating TE-based fibrosis compared with that diagnosed using a liver biopsy was similar; the diagnostic methods returned approximately the same mean ELF scores for individuals with advanced fibrosis, and the quantified scores were correlated with ELF. Another limitation is that ELF is a proprietary test that cannot be calculated using routinely collected clinical or laboratory parameters; in the context of its accuracy for estimating advanced fibrosis noninvasively, the cost-effectiveness of its use should be studied separately. Although we found no evidence for this outcome, prolonged storage time for the serum samples used for calculation of ELF scores in this study may have resulted in some sample degradation. A strength of this study is the large number of individuals with diabetes in our sample. This allowed for a subgroup analysis that may be helpful to endocrinologists and diabetologists given that the prevalence of NAFLD and NASH continue to increase in their patient populations.

## Conclusions

Among a large sample of patients with NAFLD and liver fibrosis diagnostic test results, we found that to have clinically relevant accuracy, the ELF score can be combined with an easily calculated fib-4 score. These findings, once independently validated, suggest that these NITs may be used in clinical practice to help steer treatment and decision-making for patients with NAFLD, including those with type 2 diabetes.

## Notes

Supplement. **eTable 1.** Clinical and Demographic Characteristics of Study Participants by Presence of Advanced Fibrosis **eTable 2.** Sensitivity Analysis of Enhanced Liver Fibrosis Performance for Estimating Histologic Advanced Fibrosis **eTable 3.** Comparison of Area Under the Curve Among 3 Noninvasive Tests for Advanced Fibrosis **eTable 4.** Accuracy of Estimating Advanced Fibrosis With Enhanced Liver Fibrosis Score Among Patients With and Without Type 2 Diabetes **eTable 5.** Accuracy of Estimating Advanced Fibrosis With Enhanced Liver Fibrosis Score Among Patients Aged 65 Years and Older vs Younger Than 65 Years **eTable 6.** Clinical and Demographic Characteristics of Patients in Independent Validation Sample **eFigure.** Scatterplots and Correlations of Enhanced Liver Fibrosis With Other Clinical Parameters

## References

[1] Younossi ZM, Koenig AB, Abdelatif D, Fazel Y, Henry L, Wymer M. Global epidemiology of nonalcoholic fatty liver disease—meta-analytic assessment of prevalence, incidence, and outcomes. Hepatology. 2016;64(1):73-84. doi:10.1002/hep.28431 26707365

[2] Younossi ZM, Stepanova M, Afendy M, . Changes in the prevalence of the most common causes of chronic liver diseases in the United States from 1988 to 2008. Clin Gastroenterol Hepatol. 2011;9(6):524-530.e1. doi:10.1016/j.cgh.2011.03.020 21440669

[3] Dulai PS, Singh S, Patel J, . Increased risk of mortality by fibrosis stage in nonalcoholic fatty liver disease: systematic review and meta-analysis. Hepatology. 2017;65(5):1557-1565. doi:10.1002/hep.29085 28130788 PMC5397356

[4] European Association for Study of Liver; Asociacion Latinoamericana para el Estudio del Higado. EASL-ALEH clinical practice guidelines: non-invasive tests for evaluation of liver disease severity and prognosis. J Hepatol. 2015;63(1):237-264. doi:10.1016/j.jhep.2015.04.006 25911335

[5] European Association for the Study of the Liver (EASL); European Association for the Study of Diabetes (EASD); European Association for the Study of Obesity (EASO). EASL-EASD-EASO clinical practice guidelines for the management of non-alcoholic fatty liver disease. Obes Facts. 2016;9(2):65-90. doi:10.1159/000443344 27055256 PMC5644799

[6] Chalasani N, Younossi Z, Lavine JE, . The diagnosis and management of nonalcoholic fatty liver disease: practice guidance from the American Association for the Study of Liver Diseases. Hepatology. 2018;67(1):328-357. doi:10.1002/hep.29367 28714183

[7] Rinella ME, Tacke F, Sanyal AJ, Anstee QM; participants of the AASLD/EASL Workshop. Report on the AASLD/EASL joint workshop on clinical trial endpoints in NAFLD. Hepatology. 2019;70(4):1424-1436. doi:10.1002/hep.30782 31287572

[8] Rockey DC, Caldwell SH, Goodman ZD, Nelson RC, Smith AD; American Association for the Study of Liver Diseases. Liver biopsy. Hepatology. 2009;49(3):1017-1044. doi:10.1002/hep.22742 19243014

[9] Ratziu V, Charlotte F, Heurtier A, ; LIDO Study Group. Sampling variability of liver biopsy in nonalcoholic fatty liver disease. Gastroenterology. 2005;128(7):1898-1906. doi:10.1053/j.gastro.2005.03.084 15940625

[10] Younossi ZM, Corey KE, Alkhouri N, ; US Members of the Global Nash Council. Clinical assessment for high-risk patients with non-alcoholic fatty liver disease in primary care and diabetology practices. Aliment Pharmacol Ther. 2020;52(3):513-526. doi:10.1111/apt.15830 32598051

[11] Younossi ZM, Noureddin M, Bernstein D, . Role of noninvasive tests in clinical gastroenterology practices to identify patients with nonalcoholic steatohepatitis at high risk of adverse outcomes: expert panel recommendations. Am J Gastroenterol. 2021;116(2):254-262. doi:10.14309/ajg.0000000000001054 33284184

[12] Biomarker qualification letter of intent (LOI) content elements. US Food and Drug Administration. Accessed June 4, 2021. https://www.fda.gov/media/135355/download

[13] Vali Y, Lee J, Boursier J, ; LITMUS systematic review team. Enhanced liver fibrosis test for the non-invasive diagnosis of fibrosis in patients with NAFLD: a systematic review and meta-analysis. J Hepatol. 2020;73(2):252-262. doi:10.1016/j.jhep.2020.03.036 32275982

[14] Sharma C, Cococcia S, Ellis N, Parkes J, Rosenberg W. Systematic review: accuracy of the enhanced liver fibrosis test for diagnosing advanced liver fibrosis and cirrhosis. J Gastroenterol Hepatol. 2021; Epub ahead of print. doi:10.1111/jgh.15482 33668077

[15] Staufer K, Halilbasic E, Spindelboeck W, . Evaluation and comparison of six noninvasive tests for prediction of significant or advanced fibrosis in nonalcoholic fatty liver disease. United European Gastroenterol J. 2019;7(8):1113-1123. doi:10.1177/2050640619865133 31662868 PMC6794685

[16] Anstee QM, Lawitz EJ, Alkhouri N, . Noninvasive tests accurately identify advanced fibrosis due to NASH: baseline data from the Stellar trials. Hepatology. 2019;70(5):1521-1530. doi:10.1002/hep.30842 31271665

[17] Rich NE, Oji S, Mufti AR, . Racial and ethnic disparities in nonalcoholic fatty liver disease prevalence, severity, and outcomes in the United States: a systematic review and meta-analysis. Clin Gastroenterol Hepatol. 2018;16(2):198-210.e2. doi:10.1016/j.cgh.2017.09.041 28970148 PMC5794571

[18] Kleiner DE, Brunt EM, Van Natta M, ; Nonalcoholic Steatohepatitis Clinical Research Network. Design and validation of a histological scoring system for nonalcoholic fatty liver disease. Hepatology. 2005;41(6):1313-1321. doi:10.1002/hep.20701 15915461

[19] Day J, Patel P, Parkes J, Rosenberg W. Derivation and performance of standardized enhanced liver fibrosis (ELF) test thresholds for the detection and prognosis of liver fibrosis. J Appl Lab Med. 2019;3(5):815-826. doi:10.1373/jalm.2018.027359 31639756

[20] Zobair Younossi, Ming-Lung Yu, Mohamed El Kassas, Gamal Esmat, Yusuf Yilmaz, Marlen Castellanos Fernandez, . Clinical and patient-reported outcomes data for patients with non-alcoholic fatty liver disease (NAFLD) and non-alcoholic steatohepatitis (NASH) across the world: data from the Global NASH Registry. Abstract presented at the annual American Association for the Study of Liver Diseases The Liver Meeting; November 2019; Boston, Massachusetts.

[21] Younossi ZM, Ong JP, Takahashi H, ; Global NASH Council. A global survey of physicians knowledge about non-alcoholic fatty liver disease. Clin Gastroenterol Hepatol. 2021;S1542-3565(21)00719-9. doi:10.1016/j.cgh.2021.06.048 34229038

[22] Majumdar A, Campos S, Gurusamy K, Pinzani M, Tsochatzis EA. Defining the minimum acceptable diagnostic accuracy of noninvasive fibrosis testing in cirrhosis: a decision analytic modeling study. Hepatology. 2020;71(2):627-642. doi:10.1002/hep.30846 31297832

[23] Younossi ZM, Gramlich T, Matteoni CA, Boparai N, McCullough AJ. Nonalcoholic fatty liver disease in patients with type 2 diabetes. Clin Gastroenterol Hepatol. 2004;2(3):262-265. doi:10.1016/S1542-3565(04)00014-X 15017611

[24] Kim TH, Kim SY, Tang A, Lee JM. Comparison of international guidelines for noninvasive diagnosis of hepatocellular carcinoma: 2018 update. Clin Mol Hepatol. 2019;25(3):245-263. doi:10.3350/cmh.2018.0090 30759967 PMC6759428

[25] Lim J, Singal AG. Surveillance and diagnosis of hepatocellular carcinoma. Clin Liver Dis (Hoboken). 2019;13(1):2-5. doi:10.1002/cld.761 30911378 PMC6430214

[26] Yeo YH, Hwang J, Jeong D, . Surveillance of patients with cirrhosis remains suboptimal in the United States. J Hepatol. 2021;S0168-8278(21)00307-X; Epub ahead of print. doi:10.1016/j.jhep.2021.04.042 33965477

