# Supplementary material for: Performance of the Enhanced Liver Fibrosis Test to Estimate Advanced Fibrosis Among Patients With Nonalcoholic Fatty Liver Disease
Source: JAMA Netw Open. 2021 Sep 16;4(9):e2123923. doi: 10.1001/jamanetworkopen.2021.23923 (PMC8446814; doi:10.1001/jamanetworkopen.2021.23923)
Supplement: Supplement. — eTable 1. Clinical and Demographic Characteristics of Study Participants by Presence of Advanced Fibrosis eTable 2. Sensitivity Analysis of Enhanced Liver Fibrosis Performance for Estimating Histologic Advanced Fibrosis eTable 3. Comparison of Area Under the Curve Among 3 Noninvasive Tests for Advanced Fibrosis eTable 4. Accuracy of Estimating Advanced Fibrosis With Enhanced Liver Fibrosis Score Among Patients With and Without Type 2 Diabetes eTable 5. Accuracy of Estimating Advanced Fibrosis With Enhanced Liver Fibrosis Score Among Patients Aged 65 Years and Older vs Younger Than 65 Years eTable 6. Clinical and Demographic Characteristics of Patients in Independent Validation Sample eFigure. Scatterplots and Correlations of Enhanced Liver Fibrosis With Other Clinical Parameters [file jamanetwopen-e2123923-s001.pdf]

Supplemental Online Content

Younossi ZM, Felix S, Jeffers T, et al. Performance of the enhanced liver fibrosis test to estimate advanced fibrosis among patients with nonalcoholic fatty liver disease. *JAMA Netw Open*. 2021;4(9):e2123923. doi:10.1001/jamanetworkopen.2021.23923

eTable 1. Clinical and Demographic Characteristics of Study Participants by Presence of Advanced Fibrosis

eTable 2. Sensitivity Analysis of Enhanced Liver Fibrosis Performance for Estimating Histologic Advanced Fibrosis

eTable 3. Comparison of Area Under the Curve Among 3 Noninvasive Tests for Advanced Fibrosis

eTable 4. Accuracy of Estimating Advanced Fibrosis With Enhanced Liver Fibrosis Score Among Patients With and Without Type 2 Diabetes

eTable 5. Accuracy of Estimating Advanced Fibrosis With Enhanced Liver Fibrosis Score Among Patients Aged 65 Years and Older vs Younger Than 65 Years

eTable 6. Clinical and Demographic Characteristics of Patients in Independent Validation Sample

eFigure. Scatterplots and Correlations of Enhanced Liver Fibrosis With Other Clinical Parameters

This supplemental material has been provided by the authors to give readers additional information about their work.

eTable 1. Clinical and Demographic Characteristics of Study Participants by Presence of Advanced Fibrosis

**A**	Advanced fibrosis	No advanced fibrosis	p	All
N	113	350		463
Age, years	54.0 +/- 11.5	45.8 +/- 12.2	<0.0001	47.8 +/- 12.6
Male gender	43 (38.1%)	100 (28.6%)	0.06	143 (30.9%)
Female gender	70 (61.9%)	250 (71.4%)	0.06	320 (69.1%)
White race	90 (79.6%)	234 (66.9%)	0.0099	324 (70.0%)
Black race	2 (1.8%)	56 (16.0%)	0.0001	58 (12.5%)
Asian race	4 (3.5%)	17 (4.9%)	0.56	21 (4.5%)
Hispanic race	14 (12.4%)	27 (7.7%)	0.13	41 (8.9%)
Other race	3 (2.7%)	14 (4.0%)	0.51	17 (3.7%)
Body mass index, kg/m^2	37.4 +/- 8.7	41.7 +/- 10.2	<0.0001	40.7 +/- 10.0
Type 2 diabetes	73 (64.6%)	88 (25.2%)	<0.0001	161 (34.8%)
Hyperlipidemia	66 (64.7%)	165 (53.9%)	0.06	231 (56.6%)
Hypertension	72 (69.2%)	170 (52.0%)	0.0020	242 (56.1%)
Alanine aminotransferase, U/L	55.6 +/- 44.0	44.2 +/- 39.3	0.0012	47.0 +/- 40.8
Aspartate aminotransferase, U/L	48.4 +/- 33.3	32.9 +/- 26.3	<0.0001	36.7 +/- 28.9
Platelet count, 10^9/L	204.2 +/- 74.5	270.9 +/- 68.5	<0.0001	254.3 +/- 75.7
ELF score	10.1 +/- 1.3	8.64 +/- 1.00	<0.0001	8.99 +/- 1.23
FIB-4 score	2.06 +/- 1.37	0.940 +/- 0.731	<0.0001	1.22 +/- 1.05
NAFLD Fibrosis Score (NFS)	0.206 +/- 1.472	-1.09 +/- 1.83	<0.0001	-0.765 +/- 1.833
**B**	Advanced fibrosis by TE	No advanced fibrosis by TE	p	All
N	79	383		462
Age, years	56.2 +/- 10.8	58.8 +/- 13.3	0.05	58.3 +/- 12.9
Male gender	35 (44.3%)	224 (58.5%)	0.0208	259 (56.1%)
Female gender	44 (55.7%)	159 (41.5%)	0.0208	203 (43.9%)
White race	58 (73.4%)	246 (64.2%)	0.12	304 (65.8%)
Black race	6 (7.6%)	49 (12.8%)	0.19	55 (11.9%)
Asian race	4 (5.1%)	29 (7.6%)	0.43	33 (7.1%)
Hispanic race	10 (12.7%)	33 (8.6%)	0.26	43 (9.3%)
Other race	1 (1.3%)	26 (6.8%)	0.06	27 (5.8%)
Body mass index, kg/m^2	37.3 +/- 8.1	30.9 +/- 5.7	<0.0001	31.9 +/- 6.6
Type 2 diabetes	55 (69.6%)	122 (31.9%)	<0.0001	177 (38.3%)
Hyperlipidemia	56 (70.9%)	296 (77.5%)	0.21	352 (76.4%)
Hypertension	52 (66.7%)	262 (68.6%)	0.74	314 (68.3%)
Alanine aminotransferase, U/L	55.4 +/- 35.3	38.6 +/- 30.5	<0.0001	41.5 +/- 32.0
Aspartate aminotransferase, U/L	50.3 +/- 33.9	29.8 +/- 18.0	<0.0001	33.4 +/- 22.9
Platelet count, 10^9/L	210.7 +/- 82.6	231.0 +/- 66.7	0.0265	227.5 +/- 70.0
ELF score	10.0 +/- 1.1	8.96 +/- 0.82	<0.0001	9.14 +/- 0.97
FIB-4 score	2.17 +/- 1.51	1.40 +/- 0.75	<0.0001	1.53 +/- 0.97
NAFLD Fibrosis Score (NFS)	0.115 +/- 1.611	-1.08 +/- 1.93	<0.0001	-0.851 +/- 1.927
Transient elastography, kPa	17.7 +/- 10.0	5.67 +/- 1.63	<0.0001	7.73 +/- 6.29

© 2021 Younossi ZM et al. *JAMA Network Open.*

eTable 2. Sensitivity Analysis of Enhanced Liver Fibrosis Performance for Estimating Histologic Advanced Fibrosis

	Study sample (prevalence 24%)	Independent validation sample (prevalence 26%)	Alternative scenario 1 (prevalence 15%)	Alternative scenario 2 (prevalence 5%)
**ELF cutoff = 9.8:**				
Sensitivity, %	57.5 (47.9 - 66.8)	73.8 (68.2 - 79.3)	--	--
Specificity, %	88.9 (85.1 - 92.0)	73.5 (70.2 - 76.9)	--	--
PPV, %	62.5 (54.4 - 70.0)	49.9 (44.7 - 55.1)	47.8	21.4
NPV, %	86.6 (83.9 - 89.0)	88.7 (86.1 - 91.3)	92.2	97.5
**ELF cutoff = 11.3:**				
Sensitivity, %	19.5 (12.6 - 28.0)	17.1 (12.3 - 21.8)	--	--
Specificity, %	99.1 (97.5 - 99.8)	98.2 (97.2 - 99.2)	--	--
PPV, %	88.0 (69.1 - 96.0)	77.4 (66.1 - 88.6)	80.0	54.5
NPV, %	79.2 (77.7 - 80.7)	76.8 (74.0 - 79.7)	87.5	95.9
**ELF ≥ 9.8 and FIB-4 ≥ 2.9**				
Sensitivity, %	17.9 (10.6 - 25.2)	25.1 (19.6 - 30.6)	--	--
Specificity, %	99.7 (99.1 - 100)	97.9 (96.8 - 99.0)	--	--
PPV, %	95.0 (85.5 - 100)	81.1 (72.2 - 90.0)	91.3	75.8
NPV, %	78.5 (74.5 - 82.5)	78.6 (75.8 - 81.4)	87.3	95.8
**ELF ≥ 7.2 and FIB-4 ≥ 0.74**				
Sensitivity, %	92.5 (87.4 - 97.5)	98.3 (96.7 - 100)	--	--
Specificity, %	48.7 (43.3 - 54.2)	18.3 (15.4 - 21.3)	--	--
PPV, %	37.6 (31.7 - 43.4)	30.0 (26.8 - 33.2)	24.1	8.7
NPV, %	95.1 (91.8 - 98.4)	96.9 (93.8 - 99.9)	97.4	99.2

© 2021 Younossi ZM et al. *JAMA Network Open.*

eTable 3. Comparison of Area Under the Curve Among 3 Noninvasive Tests for Advanced Fibrosis

	**Advanced fibrosis by biopsy** (prevalence 24%)		**Advanced fibrosis by transient elastography** (prevalence 17%)	
	**All patients**	**Type 2 diabetes**	**All patients**	**Type 2 diabetes**
ELF	0.81 (0.77 - 0.85)	0.78 (0.71 - 0.84)	0.79 (0.75 - 0.82)	0.80 (0.73 - 0.86)
FIB-4	0.83 (0.79 - 0.86)	0.83 (0.76 - 0.89)	0.66 (0.61 - 0.70)	0.68 (0.61 - 0.75)
NFS	0.75 (0.71 - 0.79)	0.68 (0.60 - 0.75)	0.70 (0.65 - 0.75)	0.62 (0.54 - 0.70)

© 2021 Younossi ZM et al. *JAMA Network Open.*

eTable 4. Accuracy of Estimating Advanced Fibrosis With Enhanced Liver Fibrosis Score Among Patients With and Without Type 2 Diabetes

Prevalence of advanced fibrosis in each group is shown in brackets.

	Advanced fibrosis by biopsy		Advanced fibrosis by elastography	
	**Type 2 diabetes** (prevalence 45%)	**No type 2 diabetes** (prevalence 13%)	**Type 2 diabetes** (prevalence 31%)	**No type 2 diabetes** (prevalence 8%)
Area under the ROC curve (AUC)	0.78 (0.71 - 0.84)	0.81 (0.76 - 0.85)	0.80 (0.73 - 0.86)	0.70 (0.64 - 0.75)
**ELF cutoff = 9.8:**				
Sensitivity, %	60.3 (49.1 - 71.5)	52.5 (37.0 - 68.0)	72.7 (61.0 - 84.5)	25.0 (7.7 - 42.3)
Specificity, %	86.4 (79.2 - 93.5)	89.7 (86.0 - 93.4)	75.4 (67.8 - 83.1)	88.1 (84.2 - 92.1)
PPV, %	78.6 (67.8 - 89.3)	43.8 (29.7 - 57.8)	57.1 (45.6 - 68.7)	16.2 (4.3 - 28.1)
NPV, %	72.4 (63.8 - 80.9)	92.5 (89.2 - 95.7)	86.0 (79.4 - 92.6)	92.7 (89.5 - 96.0)
**ELF cutoff = 11.3:**				
Sensitivity, %	19.2 (10.2 - 28.2)	20.0 (7.6 - 32.4)	23.6 (12.4 - 34.9)	4.2 (0.0 - 12.2)
Specificity, %	100 (95.9 - 100)	98.9 (97.6 - 100)	100 (97.0 - 100)	99.2 (98.2 - 100)
PPV, %	100 (76.8 - 100)	72.7 (46.4 - 99.1)	100 (75.3 - 100)	33.3 (0.0 - 86.7)
NPV, %	59.9 (51.9 - 67.8)	89.0 (85.4 - 92.6)	74.4 (67.7 - 81.1)	91.8 (88.7 - 95.0)
**ELF ≥ 9.8 and FIB-4 ≥ 2.9**				
Sensitivity, %	19.4 (10.3 - 28.6)	14.7 (2.8 - 26.6)	22.2 (11.1 - 33.3)	4.6 (0.0 - 13.3)
Specificity, %	100 (95.7 - 100)	99.6 (98.7 - 100)	99.2 (97.5 - 100)	99.2 (98.1 - 100)
PPV, %	100 (76.8 - 100)	83.3 (53.5 - 100)	92.3 (77.8 - 100)	33.3 (0.0 - 86.7)
NPV, %	59.2 (51.1 - 67.2)	88.9 (85.1 - 92.7)	73.4 (66.5 - 80.3)	92.1 (88.8 - 95.3)
**ELF ≥ 7.2 and FIB-4 ≥ 0.74**				
Sensitivity, %	95.8 (91.2 - 100)	85.3 (73.4 - 97.2)	92.6 (85.6 - 99.6)	86.4 (72.0 - 100)
Specificity, %	36.9 (26.6 - 47.2)	53.0 (46.6 - 59.4)	13.7 (7.5 - 19.9)	16.3 (11.7 - 21.0)
PPV, %	56.6 (47.8 - 65.4)	20.9 (14.1 - 27.6)	33.1 (25.6 - 40.6)	8.5 (4.8 - 12.1)
NPV, %	91.2 (81.6 - 100)	96.1 (92.8 - 99.5)	80.0 (62.5 - 97.5)	93.0 (85.4 - 100)

© 2021 Younossi ZM et al. *JAMA Network Open.*

eTable 5. Accuracy of Estimating Advanced Fibrosis With Enhanced Liver Fibrosis Score Among Patients Aged 65 Years and Older vs Younger Than 65 Years

Prevalence of advanced fibrosis in each group is shown in brackets.

	Advanced fibrosis by biopsy		Advanced fibrosis by elastography	
	**Age ≥ 65** (53%)	**Age < 65** (22%)	**Age ≥ 65** (12%)	**Age < 65** (20%)
Area under the ROC curve (AUC)	0.74 (0.58-0.87)	0.80 (0.76-0.84)	0.88 (0.81-0.92)	0.80 (0.75-0.85)
**ELF cutoff = 9.8:**				
Sensitivity, %	75.0 (56.0 - 94.0)	53.8 (43.6 - 63.9)	84.2 (67.8 - 100)	50.0 (37.4 - 62.7)
Specificity, %	61.1 (38.6 - 83.6)	90.4 (87.2 - 93.5)	71.3 (63.9 - 78.7)	91.7 (88.2 - 95.2)
PPV, %	68.2 (48.7 - 87.6)	61.0 (50.4 - 71.5)	28.1 (16.4 - 39.7)	60.0 (46.4 - 73.6)
NPV, %	68.8 (46.0 - 91.5)	87.5 (84.0 - 91.0)	97.1 (94.0 - 100)	88.0 (84.0 - 92.0)
**ELF cutoff = 11.3:**				
Sensitivity, %	40.0 (18.5 - 61.5)	15.1 (7.8 - 22.3)	42.1 (19.9 - 64.3)	10.0 (2.4 - 17.6)
Specificity, %	94.4 (83.9 - 100)	99.4 (98.6 - 100)	99.3 (97.9 - 100)	99.6 (98.8 - 100)
PPV, %	88.9 (68.4 - 100)	87.5 (71.3 - 100)	88.9 (68.4 - 100)	85.7 (59.8 - 100)
NPV, %	58.6 (40.7 - 76.6)	80.7 (76.9 - 84.5)	92.8 (88.7 - 96.9)	81.6 (77.1 - 86.0)
**ELF ≥ 9.8 and FIB-4 ≥ 2.9**				
Sensitivity, %	42.1 (19.9 - 64.3)	12.6 (5.7 - 19.6)	55.6 (32.6 - 78.5)	5.2 (0.0 - 10.9)
Specificity, %	93.3 (80.7 - 100)	100 (98.8 - 100)	97.9 (95.6 - 100)	100 (98.3 - 100)
PPV, %	88.9 (68.4 - 100)	100 (71.5 - 100)	76.9 (54.0 - 99.8)	100 (29.2 - 100)
NPV, %	56.0 (36.5 - 75.5)	80.0 (75.9 - 84.0)	94.6 (91.0 - 98.2)	79.9 (75.2 - 84.7)
**ELF ≥ 7.2 and FIB-4 ≥ 0.74**				
Sensitivity, %	100 (82.4 - 100)	90.8 (84.7 - 96.9)	100 (81.5 - 100)	87.9 (79.6 - 96.3)
Specificity, %	0.0 *	51.2 (45.5 - 56.8)	1.4 (0.0 - 3.3)	24.7 (19.0 - 30.4)
PPV, %	55.9 (39.2 - 72.6)	34.8 (28.6 - 41.0)	11.3 (6.4 - 16.3)	23.6 (18.0 - 29.3)
NPV, %	79.9 (75.2 - 84.7)	95.1 (91.8 - 98.4)	100 (15.8 - 100)	88.5 (80.5 - 96.5)

* Na - no negative predictions in the subgroup.

© 2021 Younossi ZM et al. *JAMA Network Open.*

eTable 6. Clinical and Demographic Characteristics of Patients in Independent Validation Sample

	N=912
Advanced fibrosis by biopsy	240 (26.3%)
Age, years	53.8 +/- 11.3
Male gender	396 (43.4%)
Female gender	516 (56.6%)
White race	611 (70.6%)
Black race	17 (2.0%)
Asian race	86 (9.9%)
Hispanic race	141 (15.5%)
Other race	57 (6.2%)
Body mass index, kg/m^2	34.0 +/- 6.2
Type 2 diabetes	503 (55.1%)
ELF score	9.59 +/- 0.99
FIB-4	1.61 +/- 1.09
NAFLD Fibrosis Score (NFS)	-0.88 +/- 1.55

© 2021 Younossi ZM et al. *JAMA Network Open.*

eFigure. Scatterplots and Correlations of Enhanced Liver Fibrosis With Other Clinical Parameters

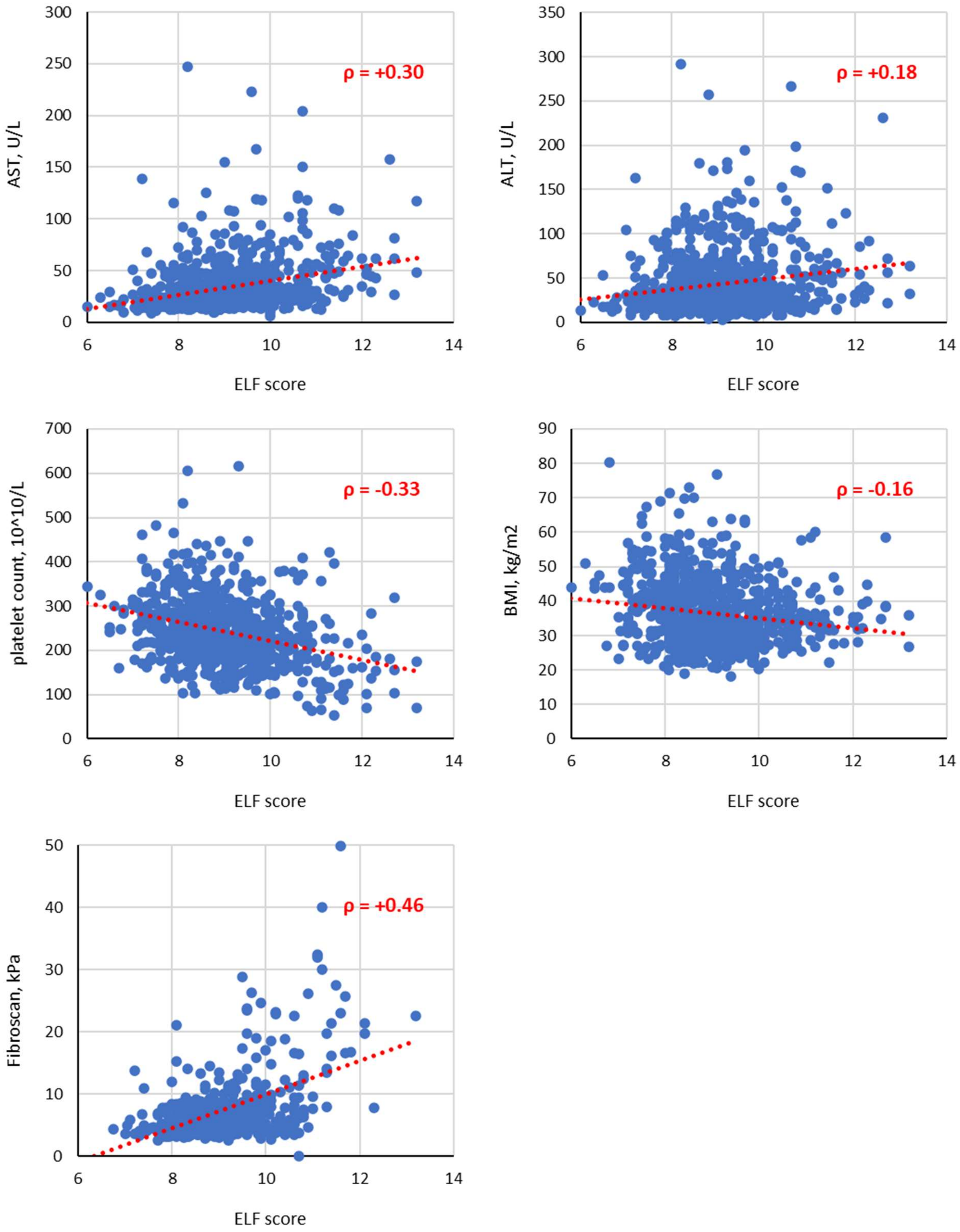

© 2021 Younossi ZM et al. *JAMA Network Open.*
